# Maternal Undernutrition during Pregnancy Alters Amino Acid Metabolism and Gene Expression Associated with Energy Metabolism and Angiogenesis in Fetal Calf Muscle

**DOI:** 10.3390/metabo11090582

**Published:** 2021-08-28

**Authors:** Susumu Muroya, Yi Zhang, Aoi Kinoshita, Kounosuke Otomaru, Kazunaga Oshima, Yuji Gotoh, Ichiro Oshima, Mitsue Sano, Sanggun Roh, Mika Oe, Koichi Ojima, Takafumi Gotoh

**Affiliations:** 1 Division of Animal Products Research, NARO Institute of Livestock and Grassland Science (NILGS), Tsukuba 305-0901, Ibaraki, Japan; mooe@affrc.go.jp (M.O.); koojima@affrc.go.jp (K.O.); 2 Department of Agricultural Sciences and Natural Resources, Kagoshima University, Korimoto 1-21-24, Kagoshima 890-8580, Japan; zhangyi439250@gmail.com (Y.Z.); k3478584@kadai.jp (A.K.); oshima@agri.kagoshima-u.ac.jp (I.O.); 3 Joint Faculty of Veterinary Medicine, Kagoshima University, Korimoto 1-21-24, Kagoshima 890-8580, Japan; otomaru@vet.kagoshima-u.ac.jp; 4 Division of Year-Round Grazing Research, NARO Western Region Agricultural Research Center, 60 Yoshinaga, Ohda 694-0013, Shimane, Japan; tenpoint@affrc.go.jp (K.O.); yujigoto@affrc.go.jp (Y.G.); 5 Faculty of Human Culture, University of Shiga Prefecture, 2500 Hassaka-cho, Hikone 522-8533, Shiga, Japan; sano.m@shc.usp.ac.jp; 6 Graduate School of Agricultural Science, Tohoku University, 468-1 Aoba, Aramaki, Aoba-ku, Sendai 980-8578, Miyagi, Japan; sanggun.roh@tohoku.ac.jp

**Keywords:** amino acid, angiogenesis, energy metabolism, fetal programming, fetal growth restriction, maternal nutrient restriction, skeletal muscle

## Abstract

To elucidate the mechanisms underlying maternal undernutrition (MUN)-induced fetal skeletal muscle growth impairment in cattle, the *longissimus thoracis* muscle of Japanese Black fetal calves at 8.5 months in utero was analyzed by an integrative approach with metabolomics and transcriptomics. The pregnant cows were fed on 60% (low-nutrition, LN) or 120% (high-nutrition, HN) of their overall nutritional requirement during gestation. MUN markedly decreased the bodyweight and muscle weight of the fetus. The levels of amino acids (AAs) and arginine-related metabolites including glutamine, gamma-aminobutyric acid (GABA), and putrescine were higher in the LN group than those in the HN group. Metabolite set enrichment analysis revealed that the highly different metabolites were associated with the metabolic pathways of pyrimidine, glutathione, and AAs such as arginine and glutamate, suggesting that MUN resulted in AA accumulation rather than protein accumulation. The mRNA expression levels of energy metabolism-associated genes, such as *PRKAA1*, *ANGPTL4*, *APLNR*, *CPT1B*, *NOS2*, *NOS3*, *UCP2*, and glycolytic genes were lower in the LN group than in the HN group. The gene ontology/pathway analysis revealed that the downregulated genes in the LN group were associated with glucose metabolism, angiogenesis, HIF-1 signaling, PI3K-Akt signaling, pentose phosphate, and insulin signaling pathways. Thus, MUN altered the levels of AAs and expression of genes associated with energy expenditure, glucose homeostasis, and angiogenesis in the fetal muscle.

## 1. Introduction

Placental insufficiency and various other maternal factors, such as environmental stress, disease, and malnutrition, contribute to intrauterine growth retardation (IUGR) during gestation, which results in impaired fetal development and decreased bodyweight (BW) at birth [1]. IUGR, also called fetal growth restriction (FGR), which leads to stunting of postnatal growth and decreased nutrient utilization efficiency in the offspring, adversely affects the whole-body composition and results in prolonged disruption of homeostasis as it predisposes the fetus to metabolic disorders, such as insulin resistance [1,2]. Maternal undernutrition (MUN) is one of the major factors that contribute to the induction of IUGR. Previous studies have reported that MUN impairs the growth and metabolism of fetal organs, including the skeletal muscle and liver [3,4,5,6,7,8], by altering the secretion and/or sensitivity of glucose, insulin, and insulin-like growth factor-1 (IGF1) [8]. The skeletal muscle accounts for 40–45% of the body mass [1] and 80% uptake of whole-body insulin-stimulated glucose. Thus, MUN-induced impairment of skeletal muscle growth and metabolism adversely affect fetal metabolism by modulating whole-body insulin sensitivity and glucose homeostasis [9].

Recently, we demonstrated that the skeletal muscle weight of fetuses of dams fed with 60% of crude protein (CP), crude fat (CF), and energy requirements was 1.27- to 1.72-fold lower than that of the fetuses of dams fed with 120% of the nutritional requirements [10]. The phenotypic alteration observed in this study promotes understanding the mechanism underlying the effect of MUN on muscle development and tissue metabolism in the fetuses. Previous studies have demonstrated that MUN from the mid-first to mid-second trimester of gestation period in sheep decreases the number of secondary fibers per fasciculi in the fetal *longissimus dorsi* and *semitendinosus* muscles [11,12]. In human fetuses diagnosed with IUGR, the DNA content decreased in the skeletal muscle at late gestation but did not show difference in protein/DNA ratios compared with healthy fetuses [13]. IUGR alters the proliferation and differentiation of myoblasts prepared from near-term sheep fetuses [14] and the proportion of type I (oxidative) and II (glycolytic) myofibers, the two major matured muscle cell types in the skeletal muscle tissue [15,16,17]. Fetal muscle growth impairment in meat animals can affect livestock production, especially meat quality parameters such as beef tenderness [18,19]. The intramuscular triglyceride content is upregulated in the nutrition-restricted fetal skeletal muscles [16]. This indicated that the skeletal muscles of nutrition-restricted fetuses are predisposed to insulin resistance by fetal programming, which can lead to intramuscular fat accumulation in beef, an important attribute for high marbled beef, such as Wagyu (Japanese Black) cattle.

In contrast to the effect on the myogenic process, the effect of MUN on metabolism of fetal skeletal muscle tissue remains poorly understood. The fetal skeletal muscle mass was reduced by MUN [10], possibly due to the retardation of whole process of skeletal muscle development. This could be a result of insufficient supply of energy and nutrient components that were required for the myogenic differentiation, muscle fiber maturation, and protein accumulation. Skeletal muscle metabolism in nutrient-restricted fetuses is hypothesized to be forced to prioritize minimal metabolism required for cellular energy and structure maintenance to sustain fetal muscle viability. Hence, metabolic pathways associated with energy metabolism and glucose homeostasis in the fetal skeletal muscle under MUN may be dysregulated with concomitant modulation in gene expression. In pigs, the concentrations of arginine, ornithine, proline, glutamine, and polyamines were markedly downregulated in the skeletal muscle under MUN [20]. The dysregulation of amino acids (AAs) and/or lipids in the muscles of the fetus with IUGR has been reported in the nutrition-restricted ewes [21] and placenta insufficiency model under high temperature and humidity in sheep [22] although the altered metabolites varied depending on the experimental conditions. MUN exerts its effect on the fetus by decreasing insulin sensitivity and promoting hypoxia [23]. Furthermore, MUN altered the gene expression profile in the fetal skeletal muscle tissue [24,25,26,27,28]. These studies have suggested that alteration of fetal skeletal muscle metabolism caused by MUN is crucial for the muscle development, however, the effect of MUN on fetal skeletal muscle metabolism has been poorly elucidated. This could be partly due to lack of experimental system leading to phenotypic alteration of fetal development by MUN, which can be overcome by setting the nutrient levels for dams to 60 and 120% as the low and high levels, respectively [10].

The effect of MUN on the fetal development is different depending on the gestational stage of cows, the extent and period of MUN, and the types of nutrient restricted [29]. In addition, difference in the result of MUN not only between animal species, breeds, or organs, but also between skeletal muscle types can be observed [24,30]. Comparison of experimental conditions among previous studies suggests that the effect of MUN on skeletal muscle gene expression varies between early and middle-to-late pregnancy of dams, between energy and protein restriction, and between levels of nutrition [24,25,26,27,28]. Notably, no significant phenotypic effect was observed in skeletal muscle mass or BW, in cases that the MUN period was limited to early-to-middle gestation [12] or that the nutritional level was compared between 70 and 100% or between 85 and 140% in requirement of energy for maintenance [30,31], even with significant effect on muscle fiber numbers and/or gene expression. Thus, the period of MUN and gestational stage of dams can alter the effect of MUN on the gene expression, metabolism, and phenotype of the fetal skeletal muscle. To elucidate the molecular basis underlying the phenotypic alteration of fetal muscle caused by MUN, an experimental design that causes significant phenotypic alteration is necessary.

This study aimed to elucidate the effect of MUN on fetal skeletal muscle metabolism that links to the retardation of the fetal muscle development in Japanese Black cows. To this end, we used a design in which the pregnant dams were fed on low-nutrition (LN) and high-nutrition (HN) diets (based on protein, fat, and energy contents) during the whole gestation (until month 8.5 post-conception) [10]. To find the phenotypic effect of MUN on fetal development under the limited knowledge regarding the MUN effect in cattle, this design is beneficial due to its marked phenotypic alteration of fetal muscles in the LN group [10]. The LN and HN groups were set to 60 and 120% of the recommended nutritional level, respectively, based on the standard diet model for prepregnant BW in the Japanese Feeding Standard for Beef Cattle (JFSBC, 2008 ed.) [32]. The changes in metabolomic and transcriptomic profiles in the fetal *longissimus thoracis* (LT) muscle were analyzed using capillary electrophoresis–time-of-flight mass spectrometry (CE-TOFMS) and microarray analysis, respectively. Bioinformatic analysis of the metabolomic and transcriptomic data was performed to understand the impact of MUN on the fetal skeletal muscle metabolism.

## 2. Results

### 2.1. Fetal Carcass Traits

To examine the effect of MUN on fetal skeletal muscle growth, the BW, LT muscle weight, and total skeletal muscle weight of half carcass (right side) were measured (Table 1). The BW, LT muscle weight, and total muscle weight of the LN group were lower than those of the HN group (*p* < 0.01), whereas the percentage of total muscle weight in BW and that of the LT muscle weight in total muscle weight were not different (*p* > 0.05). The ratios of BW, total muscle weight, and LT muscle weight in the LN group to those in the HN group were 0.72, 0.72, and 0.69, respectively. Thus, MUN markedly decreased the mass of skeletal muscles in the fetuses of the LN group.

### 2.2. Metabolomic Profile of Fetal Skeletal Muscle

To assess the effect of MUN on fetal LT muscle metabolism, the metabolomic difference was analyzed using CE-TOFMS (Table 2). In the CE-TOFMS analysis, four fetuses with the highest BW in the HN group and four fetuses with the lowest BW in the LN group were used for the analysis. In total, 169 uniquely annotated metabolites were detected (Table S1). Of these metabolites, the levels of carnosine, glutamine, glycerol, creatine, *N*^6^-methyllysine, phosphorylcholine, phenylalanine, proline, (*p* < 0.05), alanine, putrescine, creatinine, gamma-aminobutyric acid (GABA), histidine, and glycine (*p* < 0.10) in the fetal muscles of the LN group were higher than those in the fetal muscles of the HN group (Table 2). The levels of 2-aminoethylphosphonic acid (2-AEP, also called ciliatine), *myo*-inositol 2-phosphate (also called inositol 5-phosphate; Ins2P) (*p* = 0.039), and 2-hydroxyvaleric acid (2-HVA) (*p* = 0.098) in the LN fetal muscles were lower than those in the HN fetal muscles.

**Figure 1:**
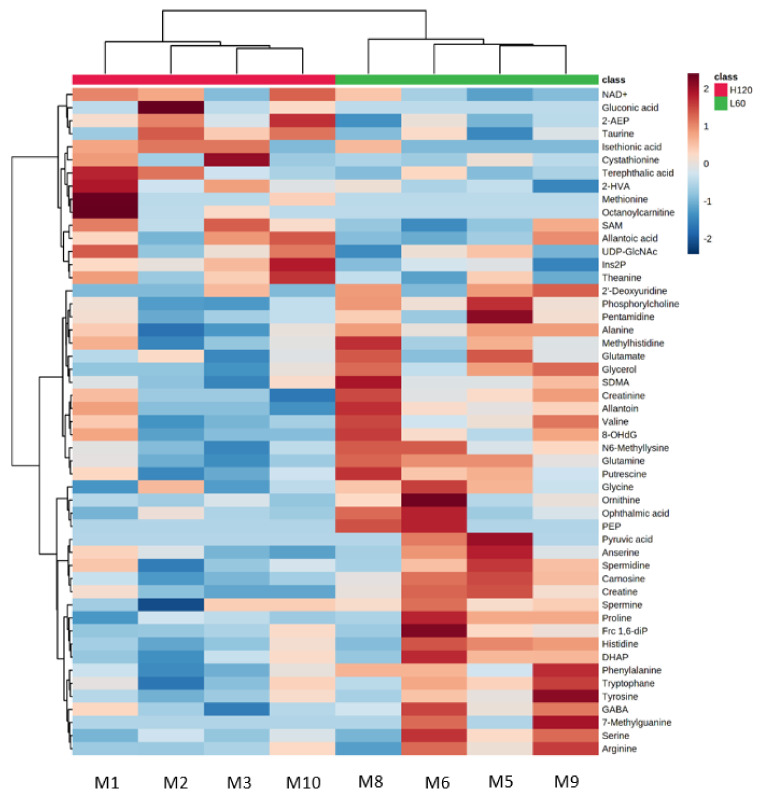
HCA using the top 50 statistically different metabolites between LN (green) and HN (red) fetuses. The row displays the metabolite and the column represents the sample. Metabolites with relatively low contents are displayed in blue, while metabolites with relatively high contents are displayed in brown. The brightness of each color corresponds to the magnitude of the difference when compared with the average value. UDP-GlcNAc: uridine diphosphate *N*-acetylglucosamine/uridine diphosphate *N*-acetylgalactosamine, 8-OHdG: 8-hydroxy-2′-deoxyguanosine, PEP: phosphoenolpyruvic acid, Frc 1,6-diP: fructose 1,6-diphosphate, DHAP: dihydroxyacetone phosphate.

Unsupervised hierarchical clustering analysis (HCA) using the top 50 significantly different metabolites between the LN and HN groups classified the fetal muscle samples into LN and HN groups. Figure 1 shows that glutamine, glycerol, phosphorylcholine, carnosine, *N*^6^-methyllysine, creatinine, putrescine, creatine, valine, and allantoin were abundant in the LN group, whereas 2-AEP, Ins2P, taurine, *S*-adenosylmethionine (SAM), UDP-*N*-acetylgalactosamine/UDP-*N*-acetylglucosamine, *N*^5^-ethylglutamine, terephthalic acid, 2-HVA, guanosine, and NAD+ were abundant in the HN group, compared with the counterpart group. This result indicated the important contribution of these metabolites to the separation of the fetal muscles between the LN and HN groups. Thus, the fetal muscle of the LN group was characterized by the abundance of metabolites such as carnosine, glutamine, glycerol, creatine, *N*^6^-methyllysine, phenylalanine, and phosphorylcholine.

To understand biological processes associated with differentially expressed metabolites between the LN and HN groups, metabolite set enrichment analysis (MSEA) was performed using the top 50 differentially expressed metabolites. The differentially expressed metabolites were significantly associated with the metabolic pathways of pyrimidine (*p* = 0.003), aminoacyl-tRNA biosynthesis (*p* = 0.007), glycerolipid (*p* = 0.007), AAs (arginine, alanine, aspartate, glutamate, histidine, and proline) (*p* < 0.05), and glutathione (*p* = 0.011) (Table 3). The metabolisms associated with glyoxylate and dicarboxylate (*p* = 0.013), and phosphonate and phosphinate (*p* = 0.017), glycerophospholipid (*p* = 0.018), galactose (*p* = 0.022), primary bile acid biosynthesis (*p* = 0.025), purine (*p* = 0.025), D-glutamine and D-glutamate (*p* = 0.029), nitrogen (*p* = 0.029), glycine, serine and threonine (*p* = 0.036), and β-alanine (*p* = 0.040) were also significantly extracted (Table 3).

### 2.3. Effect of MUN on the Gene Expression Profile of the Fetal Skeletal Muscle

**Figure 2:**
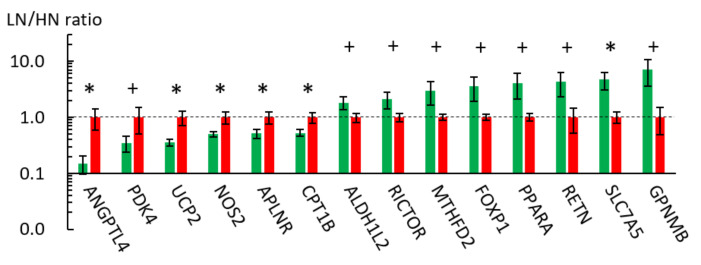
qPCR results of highly down- and upregulated fetal skeletal muscle genes in microarray analysis. The ratios of the normalized gene expression of the LN (green) to HN (red) fetuses are shown as columns. *RPL7* was used as the internal control. Error bars indicate SEM. * and + indicate differences between the LN and HN fetuses at *p* < 0.05 and *p* < 0.10, respectively. *PDK4*: pyruvate dehydrogenase kinase 4, *NOS2*: nitric oxide synthase 2, *CPT1B*: carnitine palmitoyltransferase 1B, *MTHFD2*: methylenetetrahydrofolate dehydrogenase (NADP+ dependent) 2, methenyltetrahydrofolate cyclohydrolase, *RICTOR*: RPTOR independent companion of MTOR complex 2, *FOXP1*: forkhead box P1, *PPARA*: peroxisome proliferator activated receptor alpha, *RETN*: resistin, *SLC7A5*: solute carrier family 7 member 5, *GPNMB*: glycoprotein nmb, *ALDH1L2*: aldehyde dehydrogenase 1 family member L2.

The MUN-mediated metabolic alterations in the LT muscles of the LN fetuses could result from disturbed gene expression. To investigate the association of MUN-induced metabolic alterations with gene expression, microarray-based gene expression analysis was performed using the pooled muscle RNA samples. The RNA samples were extracted from four fetuses with the highest BW in the HN group and four fetuses with the lowest BW in the LN group. Of the 12,786 detected unique genes, the expression levels of 219 genes in the fetal muscle varied > two-fold between the LN and HN groups (187 upregulated and 32 downregulated genes in the LN group). The top five downregulated genes in the LN group were retinoid X receptor α(*RXRA*), angiopoietin-like 4 (*ANGPTL4*), uncoupling protein 2 (*UCP2*), and apelin receptor (*APLNR*, also called *APJ*). Meanwhile, the top five upregulated genes in the LN group were syndecan-binding protein (*SDCBP*), olfactomedin 4 (*OLFM4*), nuclear receptor subfamily 2 group E member 3 (*NR2E3*), tribbles pseudokinase 3 (*TRIB3*), and an uncharacterized gene (XM_005208051). The downregulation of *ANGPTL4*, *UCP2*, and *ALPNR*, which are involved in energy homeostasis, in the fetuses of the LN group was validated using qRT-PCR (*p* < 0.05; Figure 2). However, the expression levels of *RXRA*, *SDCBP*, *OLFM4*, *NR2E3*, and *TRIB3* were not significantly different between the groups in the qRT-PCR analysis (*p* > 0.10).

The microarray analysis results suggested that the MUN affected energy metabolism in the fetal LT muscle of the LN group. Moreover, MSEA revealed that MUN altered the metabolic pathways associated with AAs, glutathione, nitrogen, and one-carbon cycle. Therefore, this study further focused mainly on energy metabolism-related genes that showed difference between the LN and HN groups in the microarray analysis. The qRT-PCR analysis revealed that the expression levels of carnitine palmitoyltransferase 1B (*CPT1B*) (*p* = 0.027), *NOS2* (also called inducible NOS, iNOS) (*p* = 0.024), and pyruvate dehydrogenase kinase 4 (*PDK4*) (*p* = 0.094) were downregulated, whereas those of solute carrier family 7A5 (*SLC7A5*) (*p* = 0.034), methylenetetrahydrofolate dehydrogenase/cyclohydrolase (*MTHFD2*), aldehyde dehydrogenase 1 family member L2 (*ALDH1L2*), peroxisome proliferator-activated receptor α (*PPARA*), resistin (*RETN*), glycoprotein nonmetastatic melanoma protein B (*GPNMB*), rapamycin-insensitive companion of mammalian target of rapamycin (*RICTOR*), and forkhead box protein P1 (*FOXP1*) were upregulated in the LN fetal muscles (*p* < 0.10) (Figure 2).

### 2.4. GO Analysis of MUN-Associated Metabolic Pathways

The analysis of differentially expressed genes between the LN and HN groups revealed that MUN modulated gene expression associated with energy production/consumption, NO synthesis, angiogenesis, and the metabolic pathways involved in skeletal muscle development. To understand the pathways in which the differentially expressed genes are enriched, gene ontology (GO) and the Kyoto Encyclopedia of Genes and Genomes database (KEGG) pathway analyses were performed using 3949 genes whose expression in the LN fetal muscle was >1.25-fold downregulated (Table 4) or upregulated (Table 5) when compared with those in the HN fetal muscles.

The downregulated genes in the LN fetal muscle were significantly enriched in the following KEGG pathways: glycolysis/gluconeogenesis, MAPK signaling, HIF-1 signaling, carbon metabolism, PI3K-Akt signaling, thyroid hormone signaling, pentose phosphate, insulin signaling, and p53 signaling (*p* < 0.01, Table 4). In addition, the downregulated genes were associated with GO terms, such as response to cytokine, negative regulation of transcription from RNA polymerase II promoter, angiogenesis, negative regulation of lipid catabolic process, positive regulation of blood vessel endothelial cell migration, negative regulation of protein catabolic process, as well as with those related to glycolysis/gluconeogenesis, apoptosis, and release of cytochrome c (biological process) (*p* < 0.01). Meanwhile, the upregulated genes in the LN fetal muscle were enriched in the following KEGG pathways: biosynthesis of AAs, aminoacyl-tRNA biosynthesis, glycine, serine and threonine metabolism, synthesis and degradation of ketone, and protein digestion and absorption (*p* < 0.01, Table 5). The upregulated genes were also associated with the following GO terms (biological process): one-carbon (1C) metabolic process and tetrahydrofolate metabolic process (*p* < 0.01).

**Figure 3:**
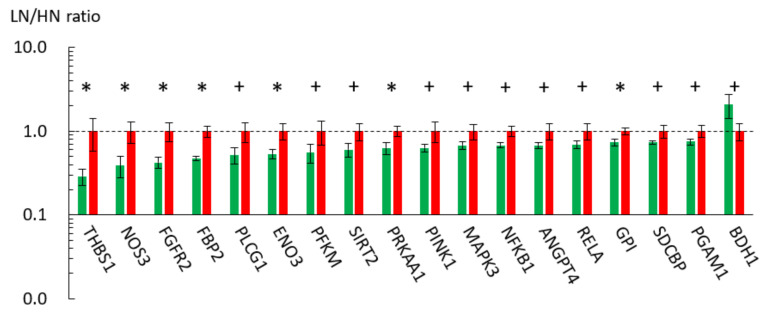
Expression of fetal skeletal muscle genes associated with significant metabolisms/pathways extracted in GO and KEGG analyses. The ratios of the normalized gene expression of the LN (green) to HN (red) fetuses are shown as columns. RPL7 was used as the internal control. Error bars indicate SEM. * and + indicate differences between the LN and HN fetuses at *p* < 0.05 and *p* < 0.10, respectively.

To validate the resultant biological processes in the GO/pathway analyses, the expression levels of the representative genes involved in these pathways or metabolisms were examined with qRT-PCR. The expression levels of genes related to glycolysis/gluconeogenesis or carbon metabolism (*GPI*, *ENO3*, *PFKM*, *FBP2*, and *PGAM1*), MAPK signaling, HIF-1 signaling, and PI3K-Akt signaling pathways (*ANGPT4*, *NOS2*, *NOS3*, *ENO3*, *MAPK3*, *NFKB1*, *PLCG1*, *PRKAA1*, *RELA*, and/or *THBS1*), pentose phosphate pathway (*GPI*, *PFKM*, and *FBP2*), and insulin signaling pathway (*FBP2* and *MAPK3*) were downregulated in the LN fetal muscle (Table 4; Figure 3). In addition, the expression level of the cellular energy sensor *PRKAA1* (cyclic AMP-activated protein kinase (AMPK) α1 subunit) was downregulated in the LN fetal muscle (*p* = 0.029), although the AMP/ATP ratio was not significantly different between the LN and HN groups based on the metabolome analysis (*p* = 0.462). Most of the downregulated genes of interest were also associated with GO terms, such as response to cytokine (*NFKB1* and *RELA*), negative regulation of transcription from RNA polymerase II promoter (*FOXP1*, *NFKB1*, and *RELA*), angiogenesis (*GPI*, *NOS3*, *ANGPT4*, and *ANGPTL4*), regulation of apoptotic process (*PINK1* and *RELA*), negative regulation of lipid catabolic process (*PRKAA1*), positive regulation of blood vessel endothelial cell migration (*ANGPTL4*, *PLCG1*, and *THBS1*), and negative regulation of protein catabolic process (*NOS2*, *SIRT2*, and *RELA*) (Table 4).

The qRT-PCR analysis also revealed that the genes associated with synthesis and degradation of ketone bodies (*BDH1*) and one-carbon metabolic process (*MTHFD2* and *ALDH1L2*) were upregulated in the LN fetal muscle (Table 5). The associated genes validated for the upregulated genes were markedly lower than those for the downregulated genes. These results indicated that the downregulated genes were associated with transcription, angiogenesis, glucose metabolism, AA metabolism, and protein catabolism, and MAPK, HIF-1, and PI3K-Akt signaling pathways.

## 3. Discussion

### 3.1. Altered Amino Acid Metabolism Was the Major Response to IUGR

In this study, MUN altered the metabolomic profile of the fetal LT muscle. Among various maternal nutritional restriction conditions, this study examined the effect of restricting global nutrition, including CP, CF, and total digestible nutrients, during whole gestation. This nutrient-restricted condition markedly decreased BW, skeletal muscle weight, and fat content in the fetuses. The weight of liver, kidney, thymus, spleen, heart, lung, rumen, omasum, and large intestine were also lower in fetuses of the LN group than in those of the HN group as reported previously [10].

**Figure 4:**
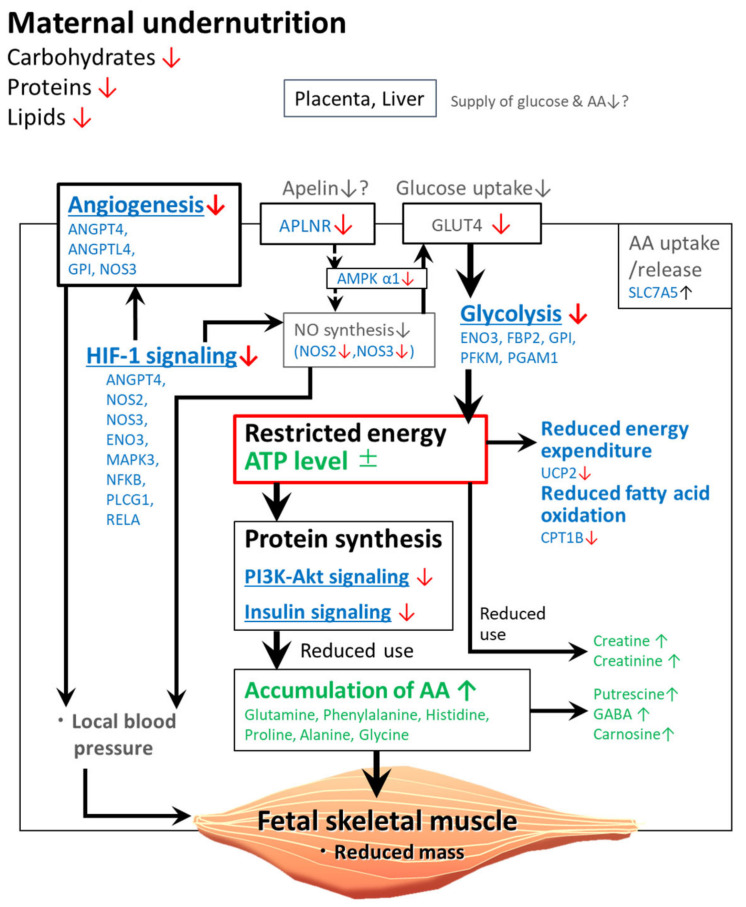
Hypothetic scheme of influences of maternal nutrient restriction on the fetal skeletal muscle molecular pathways and metabolisms. Genes and metabolites are shown in blue and green, respectively. Increase and decrease are indicated in small black and red arrows, respectively. Underlined metabolisms/pathways are significantly extracted in GO/KEGG pathway analysis. Grayed events and metabolites were not determined in this study, but some of these were confirmed in previous studies.

The retardation of fetal organs such as liver and kidney might affect the muscle growth retardation and disturbance of metabolism. In addition, MUN altered the fetal LT muscle metabolome. The levels of AAs, such as glutamine, phenylalanine, and proline in the LN fetal muscle were 1.2- to 1.5-fold higher than those in the HN fetal muscles. The increased levels of AAs, such as glutamine and alanine, were consistent with the results of MSEA, which revealed that metabolic pathways associated with arginine, alanine, aspartate, glutamate, histidine, and proline in the fetal muscles were significantly different between the LN and HN groups (Table 3). On the other hand, levels of ATP, AMP, and AMP/ATP ratio were not significantly different between the LN and HN groups. This suggests that energy balance was maintained in the LN fetal muscle to the same level as the HN fetal muscle, at the expense of energy consumption in nutrient-restricted condition. Hence, the increased AA levels in the LN fetal muscles can be attributed to the decreased utilization of AAs caused by energy insufficiency, increased AA uptake, and/or enhanced protein degradation (Figure 4).

AAs, such as glutamine and alanine, accumulate likely due to decreased protein synthesis even under optimal AA supply conditions, which can be due to restricted available energy. Hypothetically, AAs that are constitutively taken up from the circulation or generated through intracellular protein degradation accumulate in the muscle cells due to their decreased utilization in protein synthesis. This is supported by the decreased muscle mass and downregulation of genes involved in the PI3K-Akt signaling and insulin signaling pathways that activate protein synthesis [33], in the LN fetal muscles (Table 4). Similar results were also reported in previous studies, which demonstrated that the skeletal muscle mass decreased even though the AA levels were increased [22,34]. The decreased AA utilization could be associated with the downregulated expression of genes involved in the glycolytic and pentose phosphate metabolic pathways (Table 4) and decreased energy production in the LN fetal muscles. This restricted energy-mediated AA accumulation is also supported by decreased glucose uptake, which can be attributed to downregulated expression of glucose transporter 4 (GLUT4) observed previously (in submission). These changes in energy metabolism can force the fetal metabolism to prioritize skeletal muscle maintenance by saving energy rather than protein synthesis for cell growth and proliferation (Figure 4). 

In this study, the increased levels of glutamine, phenylalanine, proline, and histidine were accompanied by upregulation of *SLC7A5* (a large neutral AA transporter) in the LN fetal muscles. This suggests that the AA uptake/release is activated in the bovine fetuses of the LN group. High temperature-induced IUGR decreased the uptake of BCAAs and aminogenic AAs, and decreased the levels of metabolites, such as alanine, arginine, aspartate, and glutamate in the ovine fetal muscle [22]. These results indicate that IUGR affects the AA uptake in the skeletal muscle, although the differences in the altered AAs between the bovine and ovine models can be attributed to the experimental conditions. In addition, the contribution of protein degradation to the increase in AAs cannot be ruled out. The fetal muscle of the LN group can catabolize its proteins as evidenced by the GO analysis results that the downregulated genes were associated with the negative regulation of the protein catabolic process. In previous studies examining MUN impact on proteolytic system, the contents of calpastatin, an endogenous specific inhibitor of protease calpains, and ubiquitinated proteins were not lower in the fetal muscle under MUN, compared with those in the control fetuses [31,35]. Collectively, protein degradation is considered not to significantly contribute to the increased levels of AA in this study.

The five increased proteinogenic AAs (glutamine, proline, alanine, histidine, and glycine) in the LN fetal muscles are glucogenic, whereas phenylalanine is both glucogenic and ketogenic [36,37]. In particular, glutamine was the most abundant proteinogenic AA (3879–5444 nmol/g tissue in the fetal muscle) and is important for glucose synthesis for energy production [38,39]. Previous studies have reported that gluconeogenesis [40] and urea synthesis [41,42] are activated in the nutrient-restricted ovine fetuses. Therefore, the AAs in the LN fetal muscle may be transported to the liver and utilized for energy production through the glucose-alanine cycle.

### 3.2. Association of Altered AAs with Metabolism of Nitrogen, β-Alanine, and Glycerophospholipids

Among the increased AAs, glutamine plays important physiological roles in the synthesis of other AAs and the related metabolites [38,39]. Glutamine is a major precursor of arginine [43] that can be converted to metabolites, including NO, proline, glutamate, creatine, and polyamines, such as putrescine [38], although the content of arginine was not altered in this study. The levels of these metabolites and glutamine were increased in the LN fetal muscle, which suggested that the increased level of glutamine raised the production of these metabolites through the arginine metabolic pathways. This is consistent with the results of MSEA, which revealed that the altered metabolites in the fetuses of the LN group were mainly associated with arginine metabolism (Table 3).

Putrescine, which was one of the increased metabolites, can be generated from glutamine via ornithine. In addition, putrescine is a precursor of bioactive polyamines, spermine and spermidine. These polyamines, with their roles in the metabolism of pyrimidine and purine, are essential for cell growth, proliferation, and differentiation [44]. As MSEA revealed the enrichment of the metabolism of pyrimidine and purine (Table 3), the increased putrescine levels in the LN group can be attributed to decreased nucleotide synthesis. Similarly, the accumulation of catabolic products (proline and GABA) of arginine and glutamine can be attributed to decreased synthesis of further reaction products due to restricted energy (Figure 4).

The level of creatine, a product of arginine catabolism, was also increased. However, the increased glutamine levels may not simply have contributed to increased creatine levels in the LN fetal muscle. This is because even though creatine is abundant in the skeletal muscle, it is generated in the liver and kidney [45]. The enhanced production of creatine can be attributed to creatinine kinase-catalyzed ATP production, in which creatine is generated by transfer of phosphate from creatine phosphate to ADP [46]. Creatine catabolism may also be impaired in the fetuses of the LN group due to the shift of reaction equilibrium toward enhanced ATP production. The increased levels of creatinine, a product of muscle creatine catabolism, can result from creatine accumulation in the fetuses of the LN group (Figure 4). This is consistent with an observation of increased creatine and creatinine levels in the urine of human neonates with IUGR [47].

Carnosine (β-alanyl-L-histidine) was previously reported to be decreased in the ovine fetal muscle under restricted maternal nutrition conditions, while anserine is increased at the late gestation stage [21], which may be associated with altered histidine metabolism. The levels of carnosine and histidine were increased in the fetuses of the LN group. As the LN fetuses showed altered histidine metabolism (Table 3) and increased levels of histidine transporter SLC7A5, the enhanced histidine uptake might contribute to histidine and carnosine accumulation.

### 3.3. Other Metabolites Altered in the Fetal Muscles of the LN Group

In this study, glycerol was increased in the LN fetal muscles, which may be derived from the intake of glycerol from nutrient-restricted maternal circulation through the placenta during late gestation [48,49]. The effect of MUN on glycerol levels in the fetal muscle has not been examined. Thus, the reason for increased glycerol levels in the fetuses of the LN group is unclear. Nevertheless, pregnant ewes can produce glycerol from the triglycerides in the adipose tissues during late gestation, especially during the fasting period [48,49]. The increased glycerol level in the LN group may indicate that MUN promotes fetal muscle glycerol uptake from the pregnant dams for energy production.

Inositol phosphoglycans (IPGs), including Ins2P and *myo*-inositol (myoIns), are involved in the insulin signaling pathway [50]. The administration of IPGs to non-diabetic or diabetic rats dose-dependently alleviates hyperglycemia and promotes muscular glycogenesis [51]. Moreover, IPGs released in response to insulin receptor activation promote oxidative glucose metabolism and the tricarboxylic acid cycle [52]. Thus, Ins2P may be involved in the dysregulation of glucose metabolism in the fetuses of the LN group in this study. The fetal plasma inositol concentrations are increased in sheep and pigs with IUGR [53,54,55]. Hence, Ins2P can be a biomarker for IUGR [47,56].

The correlation between IUGR and 2-AEP or 2-HVA has not been elucidated. Rats and other higher vertebrates cannot generate 2-AEP that contains a carbon–phosphorus bond [57,58]. The ruminant protozoa can synthesize protein-bound and lipid-bound forms of 2-AEP, as well as a free form of 2-AEP [59]. Previous studies have reported that 2-AEP is detected in the goat liver, bovine brain and liver, and major tissues of rats, chicken, and humans, including skeletal muscle [59,60]. In the rat liver, 2-AEP is incorporated in phosphonolipids, such as diacylglyceryl-AEP [61], which suggests that 2-AEP is a membrane component. Thus, 2-AEP in the bovine fetus may be taken up from the pregnant cows through the placenta and utilized as a membrane component. Further studies are needed to examine the biological role of dysregulated 2-AEP in the LN fetal muscle.

Phosphorylcholine level was increased in the LN fetal muscle. Free phosphorylcholine can be generated from the catabolism of sphingomyelins as evidenced by the changes in ceramide, sphingolipid, and phospholipid metabolites in the offspring of ewes fed on a nutrient-restricted diet [21]. The roles of ceramide and sphingomyelins in the muscle are poorly understood. However, the altered phosphorylcholine level may indicate that undernutrition modulates sphingolipid signaling by promoting the production of sphingolipid-1-phosphate in the fetal skeletal muscle, which can affect not only the activation of muscle satellite cells from quiescence for postnatal muscle growth and repair [62] and also neuronal development.

*N*^6^-methyllysine is a product of protein degradation. The interaction between modified histones and altered metabolites, including methylated lysine, in fetuses with IUGR has not been completely understood. IUGR-induced metabolic alteration can be associated with alteration of histone modification. The decreased *N*^6^-acetyllysine levels in the offspring of over-fed ewes at birth suggested epigenetic changes [21]. Thus, *N*^6^-methyllysine may be released from specific lysine residues of histones in relation to altered gene expression in the fetuses of the LN group.

### 3.4. Potential Mechanisms of NOS Downregulation and Its Impact in the Fetuses of the LN Group

MUN downregulated the expression of *NOS2*, *PRKAA1*, *ANGPTL4*, *PDK4*, *APLNR*, *UCP2*, and *CPT1B*, all of which are associated with energy metabolism. NO synthase (NOS) including *NOS2* product (iNOS), plays an essential role in energy production, autoregulation of blood flow, myocyte differentiation, respiration, and glucose homeostasis of skeletal muscle [63], as well as angiogenesis of the tissue [64]. *NOS3* (endothelial NOS gene) was also downregulated in the fetuses of the LN group. To date, the effect of IUGR on NOS gene expression in the fetal tissues has not been demonstrated. On the other hand, MUN impairs NO-dependent vasodilation and increases arterial blood pressure in the ovine fetus [65]. Collectively, the present results suggest that downregulation of NOS genes is involved in impairment of the LN fetal muscle development through dysregulated blood flow and/or nutrient supply.

In this study, MUN downregulated genes participating in the HIF-1 signaling pathway (Table 4). The HIF-1 signaling pathway can be promoted by hypoxia-induced factor-α (HIF-1α) in response to inflammatory mediators, as well as hypoxia, and regulates angiogenesis and immunity through activation of genes including *NOS2* [66]. HIF-1α expression was not significantly different between the LN and HN groups (*p* = 0.142), suggesting that downregulation of *NOS2* and *NOS3* was not mediated by HIF-1α. The mechanism underlying the lower expression of HIF-1 signaling pathway genes in the LN fetuses is unclear; however, the LN fetal muscle may have reduced inflammatory activity as observed in the lower expression associated with inflammatory response (GO:0006954, *p* = 0.011, data not shown).

The downregulation of NOS genes is assumed to decrease NO synthesis and consequently can affect energy homeostasis through a decrease in the activity of AMPK, as indicated in the role for NO in the GLUT4 regulation via regulation of AMPK [67]. AMPK is a cellular energy sensor that can sense changes in energy metabolism, including glucose uptake and oxidation [68]. In addition, AMPK regulates whole-body energy homeostasis by integrating hormonal and nutritional signals from the cellular environment and the whole organism [69]. As MUN did not alter the AMP/ATP ratio, factors other than cellular energy levels could be involved in the downregulation of AMPK α1 subunit, which may be mediated by NOSs. The *NOS3* expression affects glucose uptake via GLUT4 activation in response to insulin [70]. Hence, the downregulation of *NOS3* may decrease glucose uptake, which may affect energy homeostasis (Figure 4), in coordination with the downregulated expression of glycolytic genes, such as *GPI*, *ENO3*, and *PGAM1* (Table 4).

The downregulation of NOS may also affect the muscle angiogenesis in the LN fetuses, as NO is essential for placental angiogenesis and fetal development [71]. This can be coordinated with downregulation of *ANGPTL4*, a multifunctional factor involved in metabolism and vascular homeostasis [72]. ANGPTL4 promoted NO production through the integrin/JAK/STAT3-mediated upregulation of *NOS2* expression in wound epithelia [73]. This is consistent with the results of GO analysis, which revealed that the downregulated genes were associated with angiogenesis and the relevant biological events (Table 4). Furthermore, ANGPTL4 is involved in the vascularization of the tissues in angiogenesis-related disorders [74]. The downregulated expression levels of *NOS3* and *ANGPTL4* may affect fetal muscle angiogenesis and thereby impair skeletal muscle development.

### 3.5. Downregulated Genes in the Fetuses of the LN Group Are Associated with Energy Metabolism or Angiogenesis

ANGPTL4 is involved in lipid partitioning and maintenance of lipid homeostasis [75,76], and also has a role in insulin sensitivity and glucose homeostasis [77]. Although ANGPTL4 is considered a cytokine secreted from the adipose tissue and liver, the expression of *ANGPTL4* was altered in the biceps femoris muscle of the ovine fetuses after in utero exposure to excess maternal cortisol during late gestation [78]. This suggested that ANGPTL4 regulates the fetal muscle energy metabolism, as well as lipid metabolism, by targeting the distant adipose tissue in response to the nutritional environment. The altered *ANGPTL4* expression in the ovine study was accompanied by altered *PDK4* expression [78]. PDK4, a major isoform of the pyruvate dehydrogenase kinases in the skeletal and cardiac muscles, is transcriptionally regulated by both glucocorticoids and insulin [79]. Thus, MUN dysregulates energy metabolism and glucose homeostasis partly through the downregulation of *ANGPTL4* and *PDK4*.

Previous studies have demonstrated that IUGR modulates glucose homeostasis in the adipose tissues by regulating APLN (apelin) and its receptor APLNR [80,81] but not in the skeletal muscles so far. Although the expression levels of *APLN* and *APLNR* vary depending on the tissues and developmental stages [80], they are broadly expressed in almost all adult tissues, including the skeletal muscle [82]. The apelinergic system, APLN and APLNR, are involved in regulation of glucose uptake [83] and insulin sensitivity in the soleus muscle [84], which can be mediated by AMPK [84]. Thus, the downregulated *APLNR* expression may contribute to decreased glucose uptake in the LN fetal muscles in this study. However, further studies are needed to elucidate the roles of APLNR in MUN-mediated impaired fetal muscle development.

MUN downregulated the fetal muscle expression of *UCP2* (an isoform of UCPs; known as mitochondrial transporters) that is involved in thermogenesis and energy dissipation [85]. In the human skeletal muscle, the downregulated expression of *UCP2* was associated with decreased weight loss under calorie restriction conditions [86]. This suggested that the expression of *UCP2* is downregulated under energy-depleted conditions. The fetal muscle metabolism in the LN group may shift toward energy-saving pathways by suppressing energy expenditure, such as thermogenesis due to UCP2 activity (Figure 4).

CPT1B, which is involved in the rate-limiting step of mitochondrial β-oxidation, regulates the mitochondrial uptake of long-chain acyl-CoAs [87]. MUN downregulated the CPT-1 activity in the skeletal muscle of offspring of the nutrition-restricted ewes, which may be associated with the increased fat accumulation in the offspring [16]. The findings of this study suggest that MUN downregulated the levels of *CPT1B*, which may decrease fatty acid oxidation metabolism in the fetuses of the LN group (Figure 4) due to decreased abundance of fatty acids. 

*RETN* and *GPNMB* were upregulated in the fetuses of the LN group. Previous studies have reported that RETN, an adipokine associated with insulin resistance, is downregulated in the skeletal muscle of rats fed on a high-fat and high-protein diet [88]. The upregulated expression of *GPNMB* in the blood is correlated with AMPK activation in the skeletal muscle of Zucker diabetic fatty rats [89]. Moreover, GPNMB is involved not only in hypoxic adaptation by regulating blood vessel development and energy metabolism [90] but also in adipose tissue lipogenesis [91]. These genes may contribute to altered energy metabolism in the fetuses of the LN group.

### 3.6. Altered Expression of Genes Associated with One-Carbon Cycle and Uptake of Large Neutral AAs 

Among the LN-induced upregulated genes involved in the target GO and KEGG metabolisms/pathways, the expression levels of 29 genes were examined using qRT-PCR. Of these 29 genes, the qRT-PCR analysis revealed that the expression levels of 26 genes were not significantly different between the LN and HN groups (*p* > 0.10, data not shown); only three genes showing upregulation in the microarray analysis were validated in qRT-PCR analysis (*BDH1*, *MTHFD2*, and *ALDH1L2*). Thus, the upregulation of genes varied among fetuses of the LN group compared with those of the HN group.

MTHFD2 and ALDH1L2, which are involved in folate-mediated 1C metabolism, were highly upregulated in the fetuses of the LN group (Figure 2). The 1C cycle is the crucial metabolic pathway for purine and thymidine biosynthesis, AA homeostasis, and epigenetic regulation [92]. MSEA revealed that purine metabolism and AA metabolism were activated. Glycine, an important byproduct of 1C metabolism, functions as a precursor for the biosynthesis of metabolites, including glutathione, purine, and heme [92]. The upregulated levels of glycine and MTHFD2 support the activation of AA metabolism, which may be utilized to sustain the LN fetal muscle viability.

SLC7A5, also known as large amino acid transporter 1 (LAT1), was upregulated in the fetuses of the LN group. The uptake of large neutral AAs, such as phenylalanine, leucine, and histidine, is mediated by SLC7A5 [93]. Although the mechanism of *SLC7A5* upregulation is unclear, glucose deprivation and low insulin concentration are reported to induce *SLC7A5* upregulation in the retina [94] and skeletal muscle [95], respectively. SLC7A5 also functions as an AA sensor and stimulates the mTORC1 pathway [96]. Hence, a global nutrient-restricted environment may upregulate *SLC7A5* and consequently upregulate the levels of glutamine, phenylalanine, and histidine in the LN fetal muscle. Further studies are needed to examine the mechanism underlying *SLC7A5* upregulation under nutrient-restricted conditions.

### 3.7. Levels of Nutrients for Dams to Be Compared in the Present Study

The present study lacks animals fed on 100% of the nutrient requirement. In a previous study investigating MUN effect on fetuses in cattle, no significant difference in fetal carcass was observed between the dams fed on 70 and 100% of nutritional requirements during day 30–125 of gestation [31]. This result suggested that a relatively small restriction in maternal nutrition level may not cause a significant phenotypic effect on the fetal carcass. In another study, the fetal whole-body, heart, liver, kidney, and placenta masses were not different between 85 and 140% of the required metabolic energy in the feeding for the dams [30]. No significant phenotypic effect of MUN in the latter study can be explained by the milder nutrient restriction [30]. According to their results, the nutrient level within the range of 85–140% may not be significantly different in terms of MUN effect on fetal muscle. To understand the molecular basis underlying the phenotypic effect of MUN on fetal muscle development, it was necessary to use an experimental design that causes significant phenotypic alteration. Furthermore, a broader range of nutrient levels to be compared was was needed, employing the lowest level as much as possible. The 60% was considered as a minimum level to avoid accidents of pregnant cows [32]. During the last two months of gestation, JFSBC recommends 125% and 141% levels of total digestible nutrients (TDN) and CP, respectively, when the nutrient levels required for maintenance are considered as 100%, for pregnant Japanese Black cows [32,97]. Taking these previous studies into account, we considered that 120% of the requirement was an appropriate option as a counterpart treatment to the low level of 60%. In this experimental condition, significant reduction of the masses of fetal skeletal muscles in the LN group was observed as mentioned above [10]. This enabled us to further investigate molecular mechanism of the MUN effect on fetal muscle development in the present study. Thus, we focused on 60 and 120% of the nutrient requirement to be compared; however, 100% of the requirement might be the best nutritionally standard level and is important as the control level to evaluate the nutritional effect. The MUN effect on fetal muscle development in a case using 60 and 100% levels might be different from that in a case using 60 and 120% levels. The effect of MUN, particularly on fetal skeletal muscle phenotype, needs to be further examined using levels of 60 and 100% of the nutrient requirement.

## 4. Materials and Methods

### 4.1. Animals and Dietary Treatments

This study was performed using 11 multiparous Japanese Black cattle (initial BW 488 ± 9.6 kg) at the Iriki farm of Kagoshima University and the farm of Western Region Agricultural Research Center, NARO. The animals were maintained according to the Guide for the Care and Use of Experimental Animals [98]. The experimental protocol was approved by the Kagoshima University Animal Care and Use Committee (#A18007). The management and feeding of animals were performed as described previously [10]. Briefly, the individual diets were designed for pregnant Japanese Black cows to meet 60 or 120% of energy requirement and other nutrients based on the standard diet model for prepregnant BW in JFSBC [32]. Water was supplied sufficiently. The diet comprised the formula feed, total mixed ration, and rice straw as designed previously [10]. According to the JFSC, the recommended contents of dry matter (DM), neutral detergent fiber, acidic detergent fiber, ash, CP, Ca, and *p* in diet are 68.0, 56.1, 36.0, 11.1, 8.0, 0.6, and 0.3%, respectively (all values are expressed as a percentage of DM). The cows randomly assigned to the LN (*n* = 5) and HN (*n* = 6) diet groups were fed on the respective diet during gestation including prepregnant period. Metabolizable energy of the mixture feed for 100% requirement was estimated as 8.56 MJ/kg DM. The cows were subjected to artificial insemination (AI) using male-sorted semen of an identical sire. The pregnancy was diagnosed using trans-rectal ultrasonography (HS-1500; Honda Electronics Co., Ltd., Toyohashi, Japan) at approximately day 40 post-AI.

### 4.2. Sample Collection

In total, 11 fetuses were obtained from the cows through cesarean section at the Kagoshima University Veterinary Teaching Hospital. The fetuses were euthanized by exsanguination at day 260 ± 8.3 of gestation, after injecting 200 mg of lidocaine (AstraZeneca, Osaka, Japan) into the jugular vein. Fetal BW and body length were recorded. The fetuses were dissected to collect the skeletal muscles and adipose tissues for subsequent analysis. The LT muscle was collected from the right side of the carcass, frozen with liquid nitrogen, or soaked in RNA*later*^®^ (Thermo Fisher Scientific, Tokyo, Japan), and stored at −80 °C for further analyses.

### 4.3. Sample Preparation for CE-TOFMS

Among the 11 fetuses, the fetuses with the lowest BW in the LN group (*n* = 4) and those with the highest BW in the HN group (*n* = 4) were selected. The LT muscle samples were subjected to metabolomic analysis using CE-TOFMS. The frozen muscle pieces (46.3–90.0 mg) were immediately immersed into a solution of 50% acetonitrile and 10 μM internal standard solution 1 (Human Metabolome Technologies, Tsuruoka, Japan) at 0 °C and homogenized twice at 1500 rpm for 120 s. The samples were centrifuged at 2300× *g* and 4 °C for 5 min. The upper layer solution was filtered through a Millipore 5-kDa cutoff membrane using centrifugation. The filtrate was lyophilized, suspended in Milli-Q water, and analyzed using CE-TOFMS.

### 4.4. Instrumentation and Conditions of CE-TOFMS

CE-TOFMS was performed using an Agilent capillary electrophoresis system equipped with an Agilent 6210 time-of-flight mass spectrometer, Agilent 1100 isocratic high-performance liquid chromatography pump, Agilent G1603A CE-MS adapter kit, and Agilent G1607A CE-ESI-MS sprayer kit (Agilent Technologies, Waldbronn, Germany). The analytic conditions were identical to those used in a previous study [99]. The spectrometer was scanned from *m*/*z* 50 to 1000.

### 4.5. Data Analysis of CE-TOFMS Results

The raw data of CE-TOFMS were processed with MasterHands as described in a previous study [99]. Among the detected compounds, the compounds annotated in the Human Metabolome Database (ver. 4.0, http://www.hmdb.ca/, accessed on 17 March 2021) or KEGG database (http://www.genome.jp/kegg/, accessed on 17 March 2021) were further analyzed. The relative contents of the annotated compounds were determined by comparing the peaks of compounds with the same MS properties in the analysis. To compare the relative contents of the compounds between the LN and HN groups, the peak areas were normalized to those of the internal standards (methionine sulfone for cations, (+)-camphor-10-sulfonic acid for anions) as well as to sample weight. The contents of major metabolites, such as glycolytic products, AAs, and ATP degradation products were determined using commercially available standards. The abundance of each compound used for comparative analysis was set as 0 when the level of the compound was not detected. File conversion of raw MS data, peak picking, noise reduction, and alignment of data for multiple samples were conducted as previously described [99].

### 4.6. RNA Preparation and Complementary DNA (cDNA) Synthesis

Total RNA was extracted from the LT muscle using the mirVanaTM microRNA isolation kit (Thermo Fisher Scientific, Tokyo, Japan), following the manufacturer’s instructions. The quantity and quality of the RNA were determined using an Agilent Bioanalyzer 2100 with an RNA 6000 Pico kit (Agilent Technologies, Santa Clara, CA, USA). For polymerase chain reaction (PCR) analysis, total RNA was prepared using ISOGEN II (Nippon Gene, Toyama, Japan). The cDNAs were synthesized from 1000 ng of total RNA using the ReverTra Ace qPCR RT kit (Toyobo), following the manufacturer’s instructions.

### 4.7. Microarray Analysis

Similar to metabolomic analysis, the microarray analysis was performed using the muscle samples from fetuses with the lowest BW in the LN group (*n* = 4) and those with the highest BW in the HN group (*n* = 4). The total RNA samples of four steers from the HN and LN groups were pooled and applied to a Bovine (v2) Gene Expression 4x44K Microarray (Agilent). The signals of the hybridized probes were detected using an Agilent Microarray Scanner (Agilent). The results were normalized by quantile method using GeneSpring GX (Agilent). Array data were deposited in the National Center for Biotechnology Information (NCBI) Gene Expression Omnibus (GEO) database, and are accessible through GEO Series accession number GSE176377 (http://www.ncbi.nlm.nih.gov/geo, accessed on 8 June 2021).

### 4.8. Quantitative Real-Time PCR (qRT-PCR) Analysis

The cDNA samples of the LN (*n* = 5) and HN (*n* = 6) groups were subjected to qRT-PCR analysis. The qRT-PCR analysis was performed using CFX96 thermal cycler (Bio-Rad, Hercules, CA, USA) with the QuantiTect SYBR Green PCR kit (Qiagen, Tokyo, Japan) and the primers listed in Table S2. Ribosomal protein L7 (*RPL7*) was used as an internal control. Melting curve analysis was performed to confirm the specificity of the amplification reactions.

### 4.9. Functional Annotation of Target Genes

To classify the genes of interest according to their functional annotation, GO and pathway analyses were performed on the differentially expressed genes, which were determined from qRT-PCR analysis, between the HN and LN groups. The genes of interest were analyzed using Database for Annotation, Visualization, and Integrated Discovery (version 6.7, http://david.abcc.ncifcrf.gov, accessed on 2 February 2021) with the setting of *Bos taurus* as the background species to enrich GO terms and characterize the KEGG pathway terms defined by KEGG (http://www.genome.jp/kegg/, accessed on 17 March 2021) for the respective biological process associated with the effect of MUN. The terms were considered significant at *p* < 0.05.

### 4.10. Statistical Analyses

The effects of LN and HN diets on the metabolite and gene expression profiles were determined. The data were analyzed using the two-sided Student’s *t*-test for metabolomics data or the one-sided Student’s *t*-test for PCR data based on the trend of gene expression in microarray analysis. The differences were considered significant at *p* < 0.05 or a trend at *p* < 0.10. HCA, and MSEA using KEGG as a metabolite set library were performed using MetaboAnalyst 5.0 (https://www.metaboanalyst.ca/MetaboAnalyst/faces/home.xhtml, accessed on 26 May 2021).

## 5. Conclusions

This study examined the effect of MUN on the fetal LT muscle development at the late gestational stage, the levels of AAs and other metabolites, and the expression of genes associated with energy metabolism and angiogenesis. The levels of glutamine and its related metabolites were increased, which was associated with decreased muscle mass in the fetuses of the LN group. This suggested that these AAs accumulate due to the downregulation of protein synthesis rather than the activation of protein degradation. AA accumulation may be due to restricted energy caused by the downregulation of energy homeostasis-associated genes, including *ANGPTL4*, *APLNR*, *NOS2*, and glycolytic genes, at the expense of activation of metabolisms such as fatty acids and thermogenesis. MUN also downregulated the angiogenesis-associated genes, such as *ANGPT4*, *ANGPTL4*, and *NOS3* that are important for fetal muscle development through regulation of local blood flow. The findings of this study indicate that the metabolism of AAs and the expression of genes associated with energy expenditure, glucose homeostasis, and angiogenesis were altered in the MUN fetal muscle for prioritizing cell survival over protein accumulation and muscle growth.

## Figures and Tables

**Table 1:** Phenotypic effect of maternal nutrient restriction on fetal carcass.

	HN (*n* = 5)		LN (*n* = 6)		
	Mean	SE	Mean *	SE *	*p*-Value
Age (d)	261.6	1.5	260.7	1.6	0.684
BW (kg)	32.5	0.5	23.4	2.4	0.005
Total muscle (g)	4889.4	128.7	3496.8	401.3	0.009
% of total BW	15.0	0.4	14.9	0.4	0.733
LT muscle (g)	272.0	7.4	187.0	24.1	0.008
% of total muscle	5.6	0.1	5.3	0.2	0.183

* The values of the LN group were reported previously [10].

**Table 2:** Top 20 differently expressed metabolites in fetal LT muscle between LN and HN groups *.

Compound	LN		HN		Ratio (LN/HN)	*p*-Value
	Mean	SE	Mean	SE		
Carnosine	0.0197	0.0011	0.0148	0.0007	1.3	0.006
Glutamine	0.0215	0.0009	0.0168	0.0008	1.3	0.008
Glycerol	0.1367	0.0105	0.1038	0.0072	1.3	0.022
Creatine	0.0550	0.0026	0.0447	0.0022	1.2	0.023
*N*^6^-Methyllysine	0.0037	0.0003	0.0026	0.0002	1.5	0.028
Phosphorylcholine	0.0125	0.0011	0.0089	0.0007	1.4	0.034
Phenylalanine	0.0097	0.0009	0.0066	0.0007	1.5	0.039
*myo*-Inositol 2-phosphate	0.0007	0.0000	0.0009	0.0001	0.8	0.039
2-Aminoethylphosphonic acid	0.0002	0.0000	0.0003	0.0000	0.6	0.041
Proline	0.0386	0.0033	0.0292	0.0016	1.3	0.043
Alanine	0.1565	0.0044	0.1276	0.0110	1.2	0.059
Putrescine	0.0022	0.0002	0.0015	0.0002	1.4	0.065
Creatinine	0.0189	0.0011	0.0151	0.0016	1.2	0.070
Gamma-amino butyric acid	0.0071	0.0005	0.0055	0.0005	1.3	0.077
Histidine	0.0034	0.0006	0.0019	0.0003	1.8	0.081
Glycine	0.0701	0.0065	0.0506	0.0073	1.4	0.090
2-Hydroxyvaleric acid	0.0002	0.0000	0.0002	0.0001	0.6	0.098
Taurine	0.0249	0.0019	0.0307	0.0026	0.8	0.111
*S*-Adenosylmethionine	0.0006	0.0001	0.0008	0.0001	0.7	0.113
Dihydroxyacetone phosphate	0.0082	0.0016	0.0047	0.0011	1.7	0.118

* Values in table are relative content levels (arbitrary unit). LN and HN: Low and high nutrition treatment, respectively.

**Table 3:** Top 20 fetal muscle metabolisms different between LN and HN fetuses *.

Metabolism/Pathway	Hits/Total Metabolites	*p*-Value	Increased in LN	Decreased in LN
Pyrimidine metabolism	2/39	0.003	Glutamine, Deoxyuridine	
Aminoacyl-tRNA biosynthesis	13/48	0.007	Glutamine, Phenylalanine, Proline, Alanine, Valine, Histidine, Glycine, Tryptophan, Serine, Tyrosine, Glutamate, Arginine	Methionine
Glycerolipid metabolism	2/16	0.007	Glycerol, DHAP	
Arginine biosynthesis	4/14	0.007	Glutamine, Ornithine, Glutamate, Arginine	
Alanine, aspartate and glutamate metabolism	5/28	0.010	Glutamine, Alanine, GABA, Pyruvate, Glutamate	
Glutathione metabolism	6/28	0.011	Putrescine, Glycine, Ornithine, Spermidine, Spermine, Glutamate	
Histidine metabolism	3/16	0.012	Carnosine, Histidine, Glutamate	
Arginine and proline metabolism	11/38	0.012	Creatine, Proline, Putrescine, GABA, Ornithine, Spermidine, Pyruvate, Spermine, Glutamate, Arginine	SAM
Glyoxylate and dicarboxylate metabolism	5/32	0.013	Glutamine, Glycine, Serine, Pyruvate, Glutamate	
Phosphonate and phosphinate metabolism	2/6	0.017	Phosphorylcholine	2-AEP
Glycerophospholipid metabolism	2/36	0.018	Phosphorylcholine, DHAP	
Galactose metabolism	1/27	0.022	Glycerol	
Primary bile acid biosynthesis	2/46	0.025	Glycine	Taurine
Purine metabolism	2/65	0.025	Glutamine, Allantoin	
D-Glutamine and D-glutamate metabolism	4/6	0.029	Glutamate, Glutamine	
Nitrogen metabolism	2/6	0.029	Glutamate, Glutamine	
Glycine, serine and threonine metabolism	5/33	0.036	Creatine, Glycine, Serine, Pyruvate	Cystathionine
β-Alanine metabolism	4/21	0.040	Carnosine, Histidine, Spermidine, Spermine	
Glycolysis/Gluconeogenesis	3/26	0.058	Pyruvate, PEP, DHAP	
Selenocompound metabolism	1/20	0.059	Alanine	

* Metabolisms and pathways that were extracted in MSEA using top 50 different metabolites are listed above.

**Table 4:** Fetal growth and energy associated metabolisms and pathways extracted from downregulated genes *.

Category	Term	*p*-Value	Fold Enrichment	Validated Genes
	KEGG Pathway			
	bta04940:Type I diabetes mellitus	<0.001	3.8050	
	bta00010:Glycolysis/Gluconeogenesis	<0.001	3.1407	*GPI, ENO3, PFKM, PGAM1, FBP2*
	bta04010:MAPK signaling pathway	<0.001	1.9194	*MAPK3, NFKB1, RELA*
	bta04066:HIF-1 signaling pathway	<0.001	2.4950	*ANGPT4, NOS2, NOS3, ENO3, MAPK3, NFKB1, PLCG1, RELA*
	bta01200:Carbon metabolism	<0.001	2.1974	*GPI, PGAM1, ENO3, PFKM, FBP2*
	bta04151:PI3K-Akt signaling pathway	<0.001	1.5906	*ANGPT4, NOS3, MAPK3, NFKB1, PRKAA1, RELA, THBS1*
	bta04919:Thyroid hormone signaling pathway	<0.001	2.1196	*MAPK3, PLCG1*
	bta00030:Pentose phosphate pathway	0.003	3.4713	*GPI, PFKM, FBP2*
	bta04910:Insulin signaling pathway	0.005	1.8377	*FBP2, MAPK3, PRKAA1*
	bta04115:p53 signaling pathway	0.007	2.2001	*THBS1*
	GO: Biological Process			
	GO:0006096~glycolytic process	<0.001	4.3200	*GPI, ENO3, PGAM1*
	GO:0034097~response to cytokine	<0.001	4.0909	*NFKB1, RELA*
	GO:0006094~gluconeogenesis	<0.001	4.2078	*GPI, FBP2, PGAM1*
	GO:0000122~negative regulation of transcription from RNA polymerase II promoter	0.002	1.4727	*FOXP1, NFKB1, RELA*
	GO:0001525~angiogenesis	0.002	1.9050	*GPI, NOS3, ANGPT4, ANGPTL4*
	GO:0042981~regulation of apoptotic process	0.002	1.9474	*PINK1, RELA*
	GO:0050995~negative regulation of lipid catabolic process	0.009	5.4545	*PRKAA1*
	GO:0090200~positive regulation of release of cytochrome c from mitochondria	0.009	3.6172	*PINK1*
	GO:0043536~positive regulation of blood vessel endothelial cell migration	0.010	4.2078	*ANGPTL4, PLCG1, THBS1*
	GO:0042177~negative regulation of protein catabolic process	0.010	3.1418	*NOS2, SIRT2, RELA*

* Top 20 metabolisms/pathways extracted at *p* < 0.05 are listed. GPI: glucose-6-phosphate isomerase, ENO3: enolase 3, PFKM: phosphofructokinase, muscle, FBP2: fructose-bisphosphatase 2, PGAM1: phosphoglycerate mutase 1, ANGPT4: angiopoietin 4, NOS2: nitric oxide synthase 2, NOS3: nitric oxide synthase 3, MAPK3: mitogen-activated protein kinase 3, NFKB1: nuclear factor kappa B subunit 1, PLCG1: phospholipase C gamma 1, PRKAA1: protein kinase AMP-activated catalytic subunit alpha 1, RELA: RELA proto-oncogene, NF-kB subunit, THBS1: thrombospondin 1, FOXP1: forkhead box P1, SIRT2: sirtuin 2.

**Table 5:** Fetal growth and energy associated metabolisms and pathways extracted from upregulated genes *.

Category	Term	*p*-Value	Fold Enrichment	Validated Genes
	KEGG Pathway			
	bta01230:Biosynthesis of amino acids	<0.001	2.8335	
	bta00970:Aminoacyl-tRNA biosynthesis	<0.001	3.4187	
	bta00260:Glycine, serine and threonine metabolism	<0.001	3.4636	
	bta00072:Synthesis and degradation of ketone bodies	0.001	6.4548	*BDH1*
	bta04512:ECM-receptor interaction	0.002	2.3124	
	bta00670:One carbon pool by folate	0.003	4.6021	*MTHFD2, ALDH1L2*
	bta04550:Signaling pathways regulating pluripotency of stem cells	0.003	1.9581	
	bta04974:Protein digestion and absorption	0.004	2.2541	
	GO: Biological Process			
	GO:0006730~one-carbon metabolic process	<0.001	4.4172	*MTHFD2, ALDH1L2*
	GO:0046653~tetrahydrofolate metabolic process	<0.001	10.6749	*MTHFD2*

* Top 20 metabolisms/pathways extracted at *p* < 0.05 are listed. BDH1: 3-hydroxybutyrate dehydrogenase 1, MTHFD2: methylenetetrahydrofolate dehydrogenase (NADP+ dependent) 2, methenyltetrahydrofolate cyclohydrolase, ALDH1L2: aldehyde dehydrogenase 1 family member L2.

## Data Availability

Array data were deposited in the National Center for Biotechnology Information (NCBI) Gene Expression Omnibus (GEO) database, and are accessible through GEO Series accession number GSE176377 (http://www.ncbi.nlm.nih.gov/geo, accessed on 8 June 2021).

## Supplementary Materials

The following are available online at https://www.mdpi.com/article/10.3390/metabo11090582/s1, Table S1: Relative levels of detected metabolites in LN and HN fetal LT muscle by CE-TOFMS metabolomics, Table S2: PCR primers used in this study.
